# Transcriptomic analysis of flower opening response to relatively low temperatures in *Osmanthus fragrans*

**DOI:** 10.1186/s12870-020-02549-3

**Published:** 2020-07-16

**Authors:** Jianxin Fu, Chao Zhang, Yucheng Liu, Tianhong Pang, Bin Dong, Xiaoyue Gao, Yimin Zhu, Hongbo Zhao

**Affiliations:** Zhejiang Provincial Key Laboratory of Germplasm Innovation and Utilization for Garden Plants, Key Laboratory of National Forestry and Grassland Administration on Germplasm Innovation and Utilization for Southern Garden Plants, School of Landscape Architecture, Zhejiang Agriculture and Forestry University, Hangzhou, 311300 Zhejiang P.R. China

**Keywords:** Flower development, Petal epidermal cells, Temperature, Sweet osmanthus, RNA-seq analysis

## Abstract

**Background:**

Sweet osmanthus (*Osmanthus fragrans* Lour.) is one of the top ten traditional ornamental flowers in China. The flowering time of once-flowering cultivars in *O. fragrans* is greatly affected by the relatively low temperature, but there are few reports on its molecular mechanism to date. A hypothesis had been raised that genes related with flower opening might be up-regulated in response to relatively low temperature in *O. fragrans*. Thus, our work was aimed to explore the underlying molecular mechanism of flower opening regulated by relatively low temperature in *O. fragrans*.

**Results:**

The cell size of adaxial and abaxial petal epidermal cells and ultrastructural morphology of petal cells at different developmental stages were observed. The cell size of adaxial and abaxial petal epidermal cells increased gradually with the process of flower opening. Then the transcriptomic sequencing was employed to analyze the differentially expressed genes (DEGs) under different number of days’ treatments with relatively low temperatures (19 °C) or 23 °C. Analysis of DEGs in Gene Ontology analysis showed that “metabolic process”, “cellular process”, “binding”, “catalytic activity”, “cell”, “cell part”, “membrane”, “membrane part”, “single-organism process”, and “organelle” were highly enriched. In KEGG analysis, “metabolic pathways”, “biosynthesis of secondary metabolites”, “plant-pathogen interaction”, “starch and sucrose metabolism”, and “plant hormone signal transduction” were the top five pathways containing the greatest number of DEGs. The DEGs involved in cell wall metabolism, phytohormone signal transduction pathways, and eight kinds of transcription factors were analyzed in depth.

**Conclusions:**

Several unigenes involved in cell wall metabolism, phytohormone signal transduction pathway, and transcription factors with highly variable expression levels between different temperature treatments may be involved in petal cell expansion during flower opening process in response to the relatively low temperature. These results could improve our understanding of the molecular mechanism of relatively-low-temperature-regulated flower opening of *O. fragrans*, provide practical information for the prediction and regulation of flowering time in *O. fragrans*, and ultimately pave the way for genetic modification in *O. fragrans*.

## Background

*Osmanthus fragrans* Lour. (Oleaceae) is one of the top ten traditional ornamental plants in China with more than 2500 years’ history of cultivation [1]. It is a small evergreen tree, grown as ornamental plants for its fragrant edible flowers. Cultivars of *O. fragrans* could be divided into once-flowering group and recurrent-flowering group according to different flowering habits [2]. The once-flowering cultivars bloom in autumn and the flowering time varies greatly in different areas, such as in Hangzhou, Shanghai, Nanjing, and Suzhou, or even in different years in the same area [3]. The researches on the flowering time of different cultivars indicated that relatively low temperature before blooming is the most important environmental factor determining the flower opening of *O. fragrans* [3, 4]. However, the knowledge of molecular mechanism of flower opening in *O. fragrans*, especially response to relatively low temperature, still remains limited.

In many higher plants, the growth of flower petals is the most remarkable process during flower opening. Flower petals are the most important component of reproductive organs and play vital roles in attracting the suitable pollinator(s). Flower color, size, shape and appearance, which are determined by flower petal, are important traits appreciated by the breeders and consumers [5]. One of the traits, the size of flower petal is determined by cell division at early phases of petal growth and cell expansion at later stages of flower opening [6].

Cell expansion is accompanied by a series of process including cell wall loosening, cellulose biosynthesis, polysaccharides conversion into soluble carbohydrate, ion and water uptake, and cytoskeleton rearrangement [7]. Cell wall loosening proteins include expansin (EXP), xyloglucan endotransglycosylase/hydrolase (XTH), endo-1,4-β-D-glucanase, and pectinase [8]. Among them, EXP and XTH particularly participate in disrupting the non-covalent bonds between the cellulose microfibrils and the cross-linking glycans of the cell wall to increase the cell-wall creep rate, while pectinase and endo-1,4-β-D-glucanase can degrade the cell wall [9]. In wintersweet (*Chimonanthus praecox*), the expressions of *EXP* genes increase during flower opening [10]. The developing petals of carnation show high activities of cellulase and pectin esterase [11]. These findings reveal that petal growth relevant to flower opening is probably attributed to cell expansion. Moreover, soluble carbohydrates depending on the degradation of polysaccharides can act as osmotically active compounds which could lower the osmotic water potential and facilitate water influx in order to allow cell expansion [12]. The concentration of soluble carbohydrates in the petals will increase in the flower opening process of plants such as carnation [13], rose [14], chrysanthemum [15], *Tweedia caerulea* [16], and *lisianthus* [17].

Cell expansion is regulated by both external factors, such as temperature, humidity, and the quality and quantity of light, and internal factors, such as the circadian clock and phytohormones [18, 19]. Phytohormones are the most important mediators regulating flower opening and could be affected by circadian factors or environmental factors. As far as the present researches are concerned, most of the phytohormones, such as auxin (AUX), gibberellin (GA), ethylene (ETH), brassinosteroid (BR), jasmonic acid (JA) and abscisic acid (ABA), are proven to affect flower opening [5, 6, 20–22].

In this research, potted plants of *O. fragrans* ‘Yanhong Gui’ were employed as materials to research the effects of different temperature conditions on flower opening process under controlled relatively low temperatures. Then the transcriptomic sequencing was used to analyze the differentially expressed genes (DEGs) after different number of days’ treatment under relatively high or low temperatures to figure out the key genes involved in the regulation of flower opening in relation to relatively low temperature. This study would lay foundation on fully revealing the molecular mechanism of relatively-low-temperature-regulated flower opening of *O. fragrans* and provide theoretical reference for the prediction and regulation of flowering time and genetic modification in *O. fragrans*.

## Results

### Scanning electron microscopy (SEM) and transmission electron microscopy (TEM) analysis

**Fig. 1:**
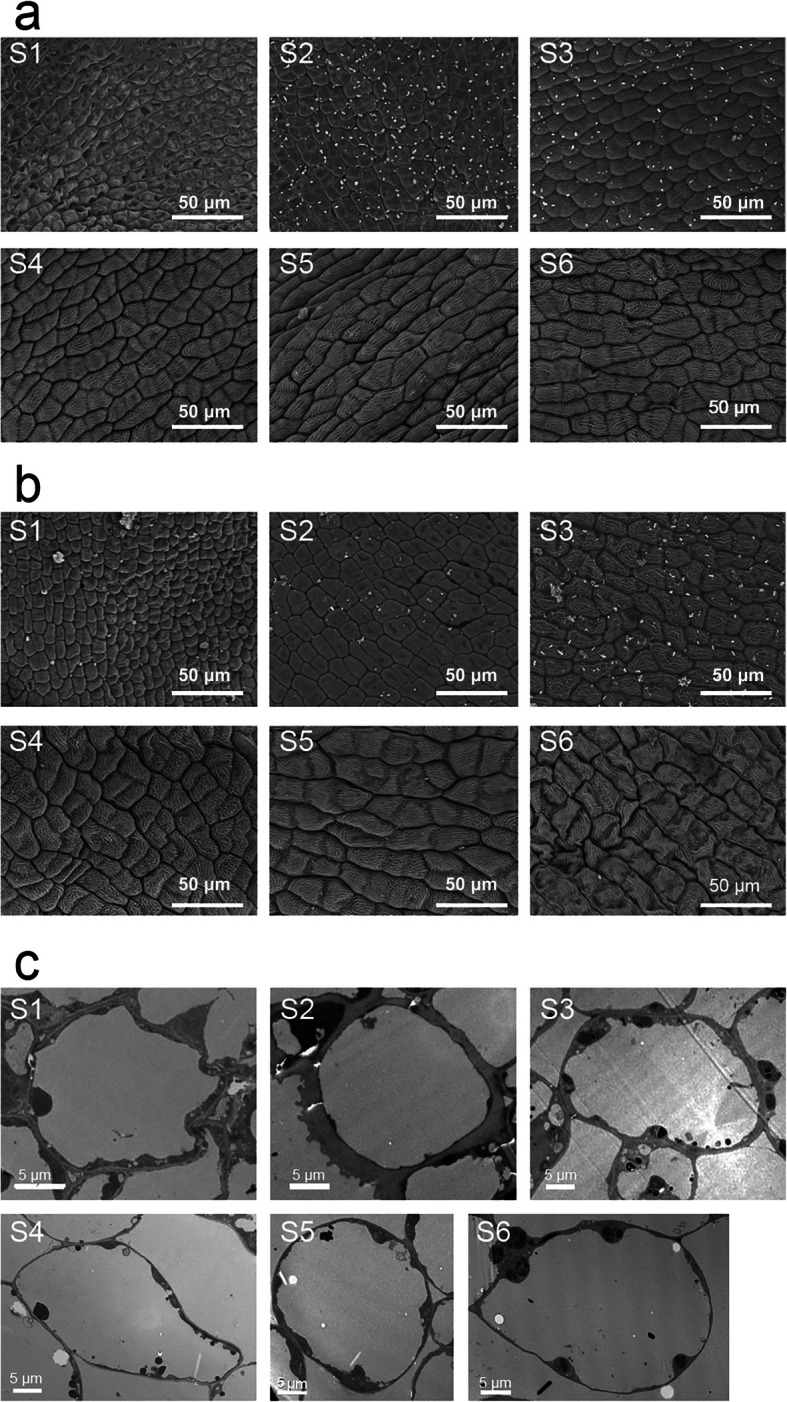
Observation on the petal cells of sweet osmanthus flower from S1 to S6 with SEM and TEM. **a** Observation on adaxial petal epidermal cells with SEM. White bar 50 μm; **b** Observation on abaxial petal epidermal cells with SEM. White bar 50 μm; **c** Observation on adaxial petal epidermal cells with TEM. White bar 5 μm. S1), the outer bud scales unfurled and the inner bud scales still furled; S2, the bud became globular-shaped and the inside bracts covering the inflorescence was visible; S3, the inflorescence burst through bracts and the florets closely crowded; S4, initial flowering stage; S5, full flowering stage; S6, pollen-scattered stage

Developmental stages of sweet osmanthus flowers for SEM and TEM analysis were described as follows: stage 1 (S1), the outer bud scales unfurled and the inner bud scales still furled; S2, the bud became globular-shaped and the inside bracts covering the inflorescence was visible; S3, the inflorescence burst through bracts and the florets closely crowded; S4, initial flowering stage; S5, full flowering stage; S6, pollen-scattered stage. Observation on the cell size of adaxial and abaxial petal epidermal cells with SEM revealed that cell size of adaxial petal epidermal cells increased gradually (*P* < 0.05, by Duncan’s multiple range test) from S1 to S6 (Fig. 1a, Table S1), while that of abaxial petal epidermal cells enlarged gradually from S1 to S5, reached the peak at S5, and then greatly decreased from S5 to S6 (Fig. 1b, Table S1). These results were coincident with results in *Gaillardia grandiflora* [23], carnation [24] and *T. caerulea* [16], indicating that the cell division ceased and cell expansion occurred during floral opening process. However, in rose [25] and *E. grandiflorum* [26] cell division and cell expansion simultaneously appeared during this process. What’s more, based on TEM observation, it was found that vacuole occupied most area of adaxial petal epidermal cells in *O. fragrans* (Fig. 1c, Table S1). The same situation occurred in *E. grandiflorum* [26]. These results indicated that the petal cell expansion was accompanied by the enlargement of vacuole. Vacuole size increased gradually from S1 to S6. At S2, vacuole size of cells was nearly two times as large as that at S1. With the cell development, vacuole size of cells at S6 was just one time larger than that at S2 (Fig. 1c, Table S1), suggesting that the growth rate from S1 to S2 was the highest during flower development.

### Bud appearance with treatments

Before 23 °C or 19 °C treatment, the buds of all plants were at S1, and they were spindle-shaped, with unfurled outer bud scales and furled inner bud scales. Bud appearance remained the same under 23 °C treatment for 2 d, 4 d, and 6 d (H2, H4, H6), as well as the 19 °C treatment for 2 d and 4 d (L2 and L4). When treated with 19 °C treatment for 6 d (L6), the bud stage reached at S2, suggesting that the relatively low temperature, like 19 °C, helps ‘Yanhong Gui’ blossom in experimental condition.

### Illumina sequencing and de novo assembly of sequence reads

To reveal the effect of 19 °C on the transcriptomic change in ‘Yanhong Gui’, we collected three biological replicate samples of H2, H4, H6, L2, L4, and L6 for reference transcriptome sequencing and RNA-seq analysis. Reference transcriptome sequencing generated 164,753,984 raw reads and 110,307,366 clean reads (Table S2). A total of 152,247 transcripts with average length of 751 nt were obtained and 96,920 unigenes with average length of 873 nt were yielded after all clean reads were assembled (Table S3).

### Functional annotation and classification

The assembled unigenes were aligned to Non-Redundant Protein Sequence Database (NR), Nucleotide Sequence Database (NT), Swiss-Prot Database, Kyoto Encyclopedia of Genes and Genomes (KEGG) Database, Eukaryotic Orthologous Groups of proteins (KOG) Database, Gene Ontology (GO) Database, and InterPro Database, in order to obtain the putative annotations (Table S4). Totally, 61,654 (63.61%) unigenes were successfully annotated using at least one database, while 8150 (8.41%) unigenes were annotated using all seven databases. Total 57,721 (59.56%) of unigenes were annotated using NR database, 59.26% of which have high homology to sequences from *Sesamum indicum* (Fig. S1). In total, 37,284 unigenes were matched in the Swiss-Prot database, which is about 38.47% of all annotated unigenes (Table S4). There were 40,764 unigenes mapped into 134 KEGG pathways which can be divided into six large pathways including “cellular processes”, “environmental information processing”, “genetic information processing”, “human diseases”, “metabolism”, and “organismal systems” (Table S5). The pathways with the highest numbers of unigenes were “metabolic pathways” (Ko01100, 8561 unigenes, 21%), followed by “biosynthesis of secondary metabolites” (Ko01110, 4440 unigenes, 10.89%), “plant-pathogen interaction” (Ko04626, 1876 unigenes, 4.6%), and “plant hormone signal transduction” (Ko04075, 1444 unigenes, 3.54%) (Table S5). Next, GO analysis was performed and a total of 16,014 unigenes (16.52% of all annotated unigenes) were categorized into 53 GO terms under three main categories: biological process, cellular component, and molecular function (Fig. S2). Proteins related to “metabolic process”, “cellular process”, and “single-organism process” were enriched in biological processes. The “single-organism process” category defines a biological process involving only one organism, and the 4806 unigenes annotated as such may be related to flowering organisms specifically. In the cellular component category, the “cell”, “cell part”, and “membrane” were the most highly presented GO terms. Under the molecular function category, the “catalytic activity”, and “binding proteins” were the most enriched (Fig. S2). We also performed KOG analysis to evaluate the functions of the assembled unigenes and 43,496 unigenes were assigned 25 KOG classification (Fig. S3). Among the 25 KOG classification, the cluster of “general function prediction only” (11,753 unigenes, 27.02%) represented the largest group, followed by “signal transduction mechanisms” (6000 unigenes, 13.79%) and “function unknown” (4761, 10.95%). “Cell motility” has the smallest proportion, only accounted for 0.20% (Fig. S3). Additionally, 46,405 (47.88%) unigenes in the transcriptome library were annotated against InterPro database (Table S4).

### RNA-sequencing (RNA-seq) and mapping to the reference transcriptome database

**Fig. 2:**
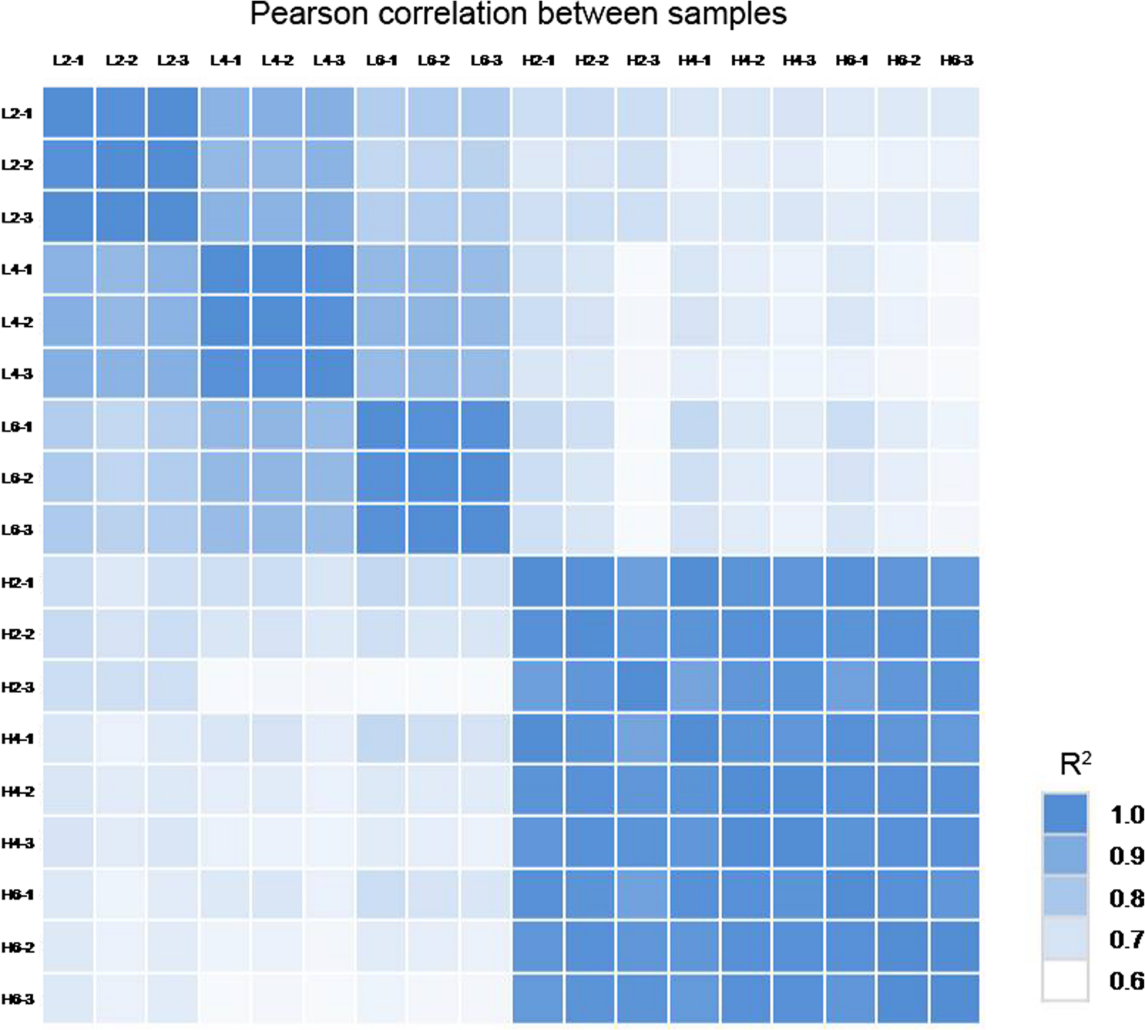
Pearson correlation analysis between samples. L2, L4 and L6 respectively represents the samples from the plants under 19 °C treatment for 2 d, 4 d, and 6 d; H2, H4 and H6 respectively represents the samples from the plants under 23 °C treatment for 2 d, 4 d, and 6 d

Eighteen cDNA libraries for RNA-seq analysis were respectively sequenced, generating 23.58–24.14 Mb raw reads (Table S2). Furthermore, we obtained 23.58–24.13 Mb clean reads in 18 cDNA libraries, with the clean data rate of 99.94–99.97%. Then, these clean reads were respectively mapped to the reference transcriptome database. Total mapped reads percentage and unique match percentage in the 18 libraries were similar. Total mapped reads percentage ranged from 86.39 to 88.44%, and unique match percentage ranged from 53.7 to 56.29% (Table S6). The correlation coefficients among biological replications are greater than 0.94 in all samples (Fig. 2, Table S7).

### Screening and analysis of DEGs

**Fig. 3:**
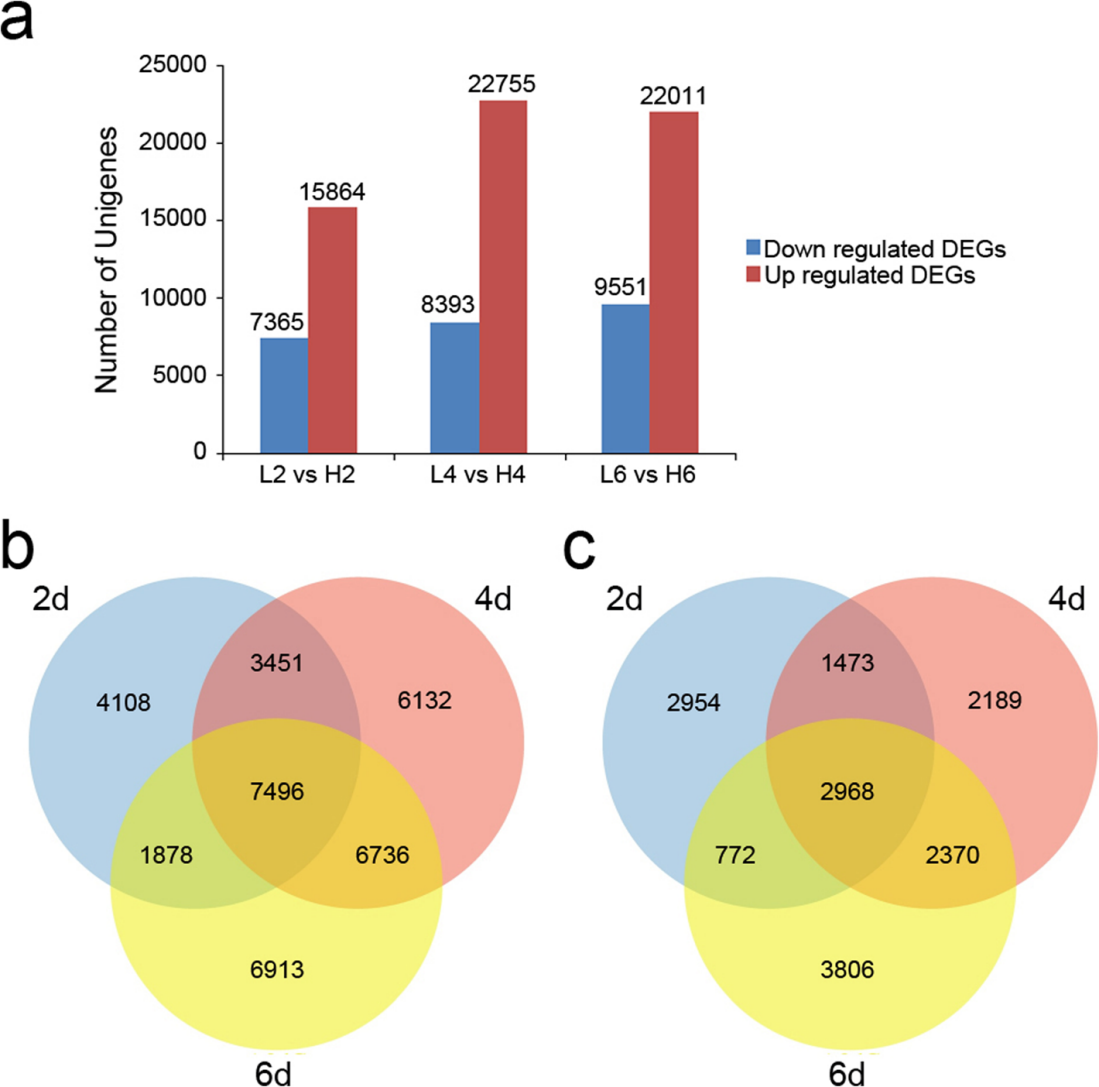
Comparative analysis of DEGs under different temperatures treated by different days. **a** The number of up and down-regulated DEGs between the three comparisons (L2 vs H2, L4 vs H4, and L6 vs H6). **b** Venn diagram of the number of up-regulated DEGs between the three comparisons. **c** Venn diagram of the number of down-regulated DEGs between the three comparisons. L2, L4 and L6 respectively represents the samples from the plants under 19 °C treatment for 2 d, 4 d, and 6 d; H2, H4 and H6 respectively represents the samples from the plants under 23 °C treatment for 2 d, 4 d, and 6 d

We performed a detailed comparative analysis of the DEGs (*P* value ≤0.01 and |log_2_Ratio| ≥ 1) in three comparisons including L2 vs H2, L4 vs H4, and L6 vs H6. In the L2 vs H2 comparison, there were 7365 and 15,864 unigenes were down-regulated and up-regulated, respectively. Meanwhile, 8393 and 22,755 unigenes were down-regulated and up-regulated in the L4 vs H4 combination, respectively. In the L6 vs H6 comparison, 9551 unigenes were down-regulated and 22,011 unigenes were up-regulated, respectively (Fig. 3a). In total, there were more up-regulated unigenes than down-regulated unigenes during floral opening under relatively low temperature. Additionally, 7496 up-regulated DEGs were shared by three comparisons, while 4108, 6132, and 6913 up-regulated DEGs were only up-regulated in the L2 vs H2, L4 vs H4, and L6 vs H6 comparison, respectively (Fig. 3b). What’s more, 2968 down-regulated DEGs were shared by three comparisons, while 2954, 2189, and 3806 down-regulated unigenes were only down-expressed in the L2 vs H2, L4 vs H4, and L6 vs H6 comparison, respectively (Fig. 3c).

### GO enrichment and KEGG pathway analysis of DEGs

In this work, GO analysis were used to classify the functions of the annotated DEGs under different temperatures treated for different number of days. In all comparisons, the most significantly enriched GO terms in biological process were “metabolic process”, “cellular process”, and “single-organism process”. In cellular component, five GO terms including “cell”, “cell part”, “membrane”, “membrane part”, and “organelle” were highly enriched. “Binding” and “catalytic activity” which were in the molecular function were significantly enriched in all comparison’s groups (Table 1). All DEGs were mapped to KEGG pathways in order to investigate the major pathways of them (Table 2). They were enriched in 132, 133, and 134 KEGG metabolic pathways in the L2 vs H2, L4 vs H4, and L6 vs H6 comparisons. In all the comparison group, “metabolic pathways”, “biosynthesis of secondary metabolites”, “plant-pathogen interaction”, “starch and sucrose metabolism”, and “plant hormone signal transduction” were the top five pathways with the largest number of DEGs (Table 2).

**Table 1:** The top ten most enriched GO terms of DEGs in all comparison groups

GO term hierarchy 1	GO term hierarchy 2	Number of DEGs		
		L2 vs H2	L4 vs H4	L6 vs H6
Biological process	metabolic process	1823	2328	2539
	cellular process	1693	2190	2331
	single-organism process	1117	1424	1556
Cellular component	cell	1287	1626	1729
	cell part	1271	1610	1710
	membrane	1162	1395	1613
	membrane part	886	1052	1229
	organelle	866	1117	1187
Molecular function	binding	1628	2135	2266
	catalytic activity	1693	2136	2379

Note: L2, L4 and L6 respectively represents the samples from the plants under 19 °C treatment for 2 d, 4 d, and 6 d; H2, H4 and H6 respectively represents the samples from the plants under 23 °C treatment for 2 d, 4 d, and 6 d

**Table 2:** The top ten most enriched KEGG of DEGs in all comparison groups

Pathway	ID	L2 vs H2		L4 vs H4		L6 vs H6	
		Percent (%)	*P*-value	Percent (%)	*P*-value	Percent (%)	*P*-value
Metabolic pathways	ko01100	22.94	3.69895E-07	23.64	2.02306E-16	23.78	9.35908E-20
Biosynthesis of secondary metabolites	ko01110	12.43	1.83429E-07	13.09	8.08594E-19	13.23	3.08193E-23
Plant-pathogen interaction	ko04626	5.42	2.90029E-05	5.43	4.82036E-07	5.67	1.43759E-11
Starch and sucrose metabolism	ko00500	4.03	9.30016E-08	3.69	3.81391E-05	3.77	8.46098E-07
Plant hormone signal transduction	ko04075	3.96	0.01001351	4.27	6.50429E-07	4.65	2.91758E-15
Protein processing in endoplasmic reticulum	ko04141	3.04	0.03015519	2.82	0.2699489	2.63	0.827178
Carbon metabolism	ko01200	2.84	0.1309717	2.87	0.06273836	3.1	0.000191297
Endocytosis	ko04144	2.72	0.5626473	2.93	0.07581657	2.73	0.5247731
Ribosome	ko03010	2.76	0.006505746	2.94	4.06865E-06	–	–
Phenylpropanoid biosynthesis	ko00940	2.55	2.74168E-13	–	–	–	–
Amino sugar and nucleotide sugar metabolism	ko00520	2.51	0.007218067	2.66	1.27859E-05	2.46	0.003741373
Biosynthesis of amino acids	ko01230	2.45	0.8067985	2.66	0.272135	2.78	0.04693815
Spliceosome	ko03040	–	–	2.82	0.8755484	2.52	0.9998826
RNA transport	ko03013	–	–	–	–	2.56	0.997384

Note: L2, L4 and L6 respectively represents the samples from the plants under 19 °C treatment for 2 d, 4 d, and 6 d; H2, H4 and H6 respectively represents the samples from the plants under 23 °C treatment for 2 d, 4 d, and 6 d

### Identification of DEGs involved in the cell wall metabolism

**Fig. 4:**
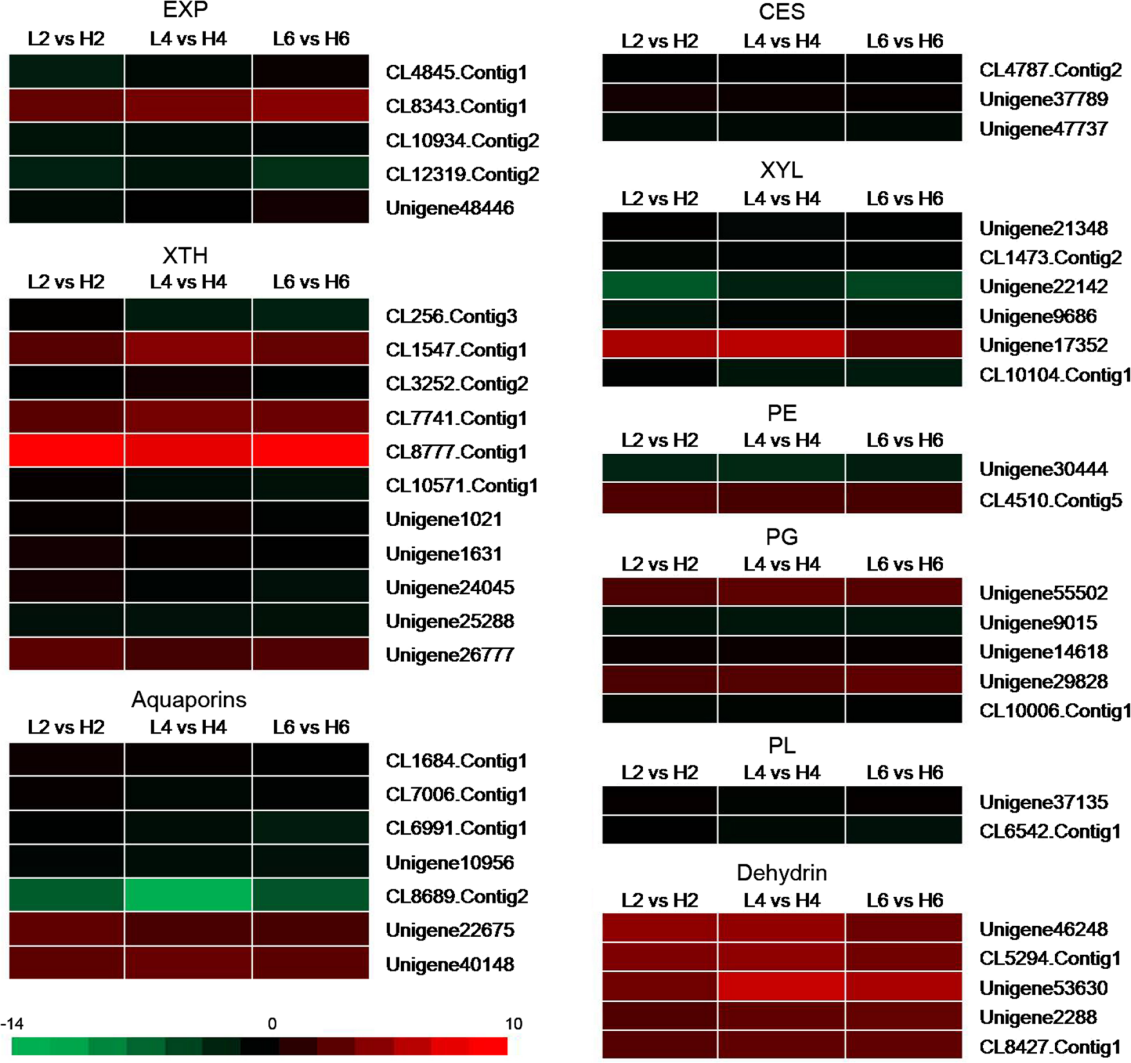
Heat maps of cell wall metabolism related genes between the three comparisons (L2 vs H2, L4 vs H4, and L6 vs H6). Red rectangles represent the up-regulated unigenes, and green rectangles represent the down-regulated unigenes. All information for each gene list can be found in Table S8. XTH: xyloglucan endotransglucosylase/hydrolase; XYL: xylosidase; CES: cellulose synthase; PE: pectinesterase; PG: polygalacturonase; PL: pectate lyase. L2, L4 and L6 respectively represents the samples from the plants under 19 °C treatment for 2 d, 4 d, and 6 d; H2, H4 and H6 respectively represents the samples from the plants under 23 °C treatment for 2 d, 4 d, and 6 d

The expansion of petal cells depends on the cell wall loosening and cellulose biosynthesis, soluble carbohydrate allocation, ion and water transport, and cytoskeleton rearrangement [7]. Therefore, we screened the DEGs which may be involved in the cell wall metabolism under different temperatures. EXP and XTH, are two kinds of essential proteins involved in cell expansion, loosening and rearranging the cell wall fibers in growing tissues [8]. In cucumber, EXPs consist of four sub families: α-EXP, β-EXP, EXP-like A and EXP-like B and were first discovered to loosen cell walls in pH dependent manner [27]. In our study, four α-EXP and one β-EXP were significantly different expressed under 23 °C and 19 °C comparisons (Fig. 4, Table S8). Among them, four *α-EXP* were up-regulated under relatively low temperature (19 °C) and one *β-EXP* was down-regulated under the treatment of 19 °C.

Eleven unigenes which encoded as XTH protein were significantly expressed, seven of them (CL256.Contig3, CL3252.Contig2, CL10571.Contig1, Unigene1021, Unigene1631, Unigene24045, and Unigene25288) were up-regulated under the treatment of 19 °C and four (CL1547.Contig1, CL7741.Contig1, CL8777.Contig1, and Unigene26777) of them were down-regulated under 19 °C treatment (Fig. 4, Table S8).

The expression of unigenes involved in cell wall synthesis, modification or hydrolysis which probably participate in the process of flower opening were also assessed (Fig. 4, Table S8). There were three cellulose synthase (CES) genes, six xylosidase (XYL) genes, two pectin esterase (PE) genes, five polygalacturonase (PG) genes, and two pectate lyase (PL) genes with dramatic fold change in all three comparisons (Fig. 4, Table S8). The expression of all CESs and PLs genes increased when treated with relatively low temperature of 19 °C for different number of days. Most of the XYLs were up-regulated under relatively low temperatures except Unigene17352. Three *PGs*’ expression increased and two of them decreased when exposed to relatively low temperature. One PE gene was greatly up-regulated while one PE gene was down-regulated when the plants were exposed to 19 °C. Several genes associated with cell wall synthesis, modification or hydrolysis are up-regulated, suggesting that these genes may play vital roles in regulating petal cell expansion.

Aquaporins can facilitate the passage of water and/or small neutral solute fluxes across membranes and can be classified into four subclasses such as plasma membrane intrinsic proteins (PIPs), tonoplast intrinsic proteins (TIPs), nodulin-26-like intrinsic membrane proteins (NIPs) and small basic intrinsic proteins (SIPs) based on the sequence homology and cellular localization [28]. Three kinds of aquaporins including two PIPs, two TIPs and three NIPs, were found in our transcriptomes and most of them were significantly up-regulated under relatively low temperature (Fig. 4, Table S8). However, only the expression level of two NIPs (Unigene22675, and Unigene40148) decreased under relatively low temperature. Dehydrins are hydrophilic, thermostable stress proteins and some of them were identified as osmotic stress-responsive protein which could facilitate the inflow and outflow of water [29]. In our research, all the dehydrin genes were down-regulated when exposed to relatively low temperature (Fig. 4, Table S8).

### Identification of DEGs involved in the phytohormone signal transduction pathway

**Fig. 5:**
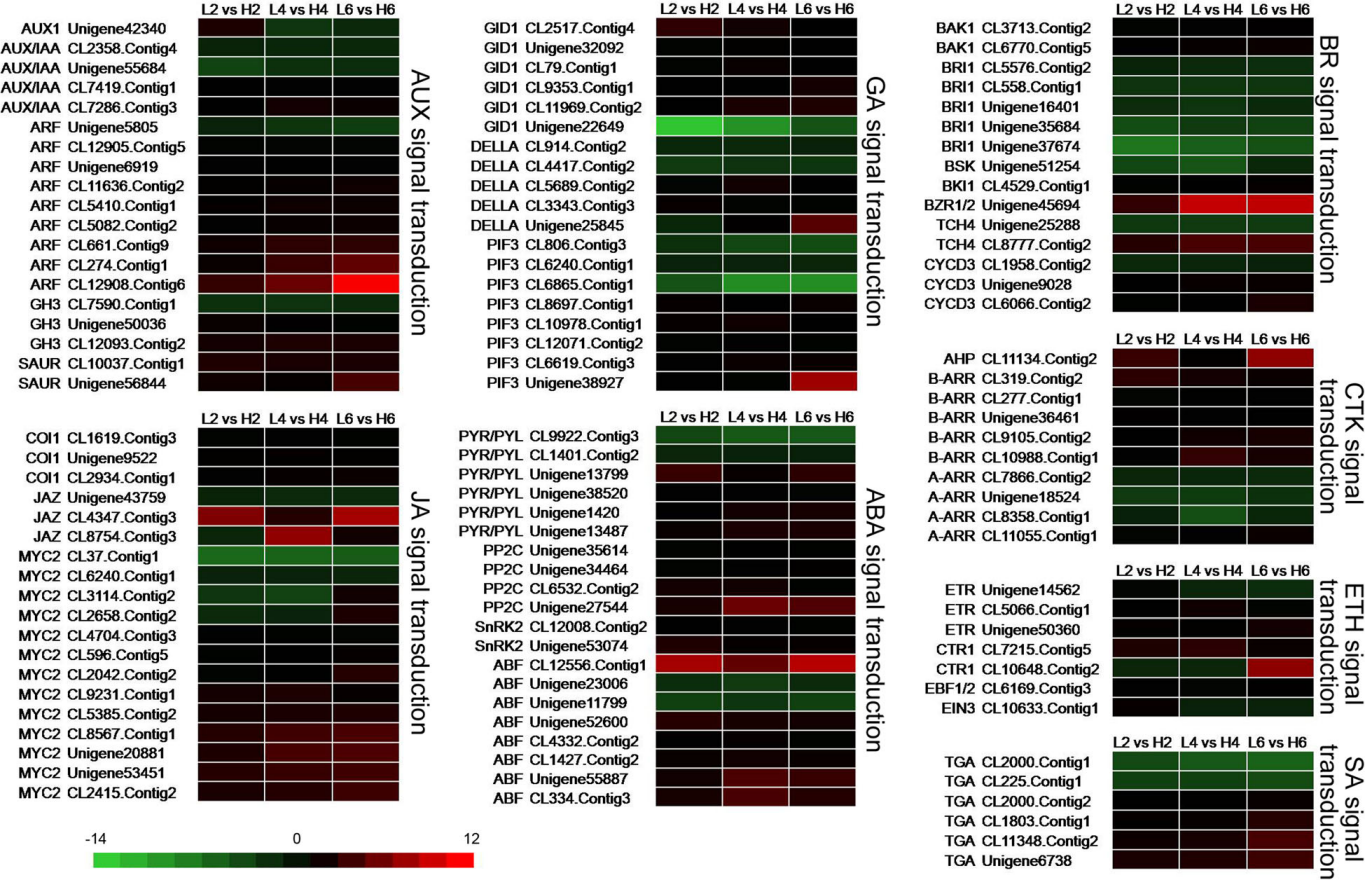
Heat maps of phytohormone signal transduction genes between the three comparisons (L2 vs H2, L4 vs H4, and L6 vs H6). Red rectangles represent the up-regulated unigenes, and green rectangles represent the down-regulated unigenes. All information for each gene list can be found in Table S9. L2, L4 and L6 respectively represents the samples from the plants under 19 °C treatment for 2 d, 4 d, and 6 d; H2, H4 and H6 respectively represents the samples from the plants under 23 °C treatment for 2 d, 4 d, and 6 d

There are some reports on phytohormones that can affect the floral opening [19], and therefore, we explored the DEGs involved in eight phytohormone signal transduction pathways. The levels of endogenous AUX were much higher in the basal petals and application of exogenous indole-3-butyric acid (IBA) can enlarge the cell length in chrysanthemum [5]. Many unigenes involved in AUX signal transduction pathway were increasingly expressed with the flower opening of rose, suggesting that AUX signaling participated in petal development [30]. In this study, nineteen unigenes were significantly differently expressed under different temperatures in AUX signal transduction pathway which included the auxin transporter protein (AUX1), auxin-induced protein/auxin-responsive protein (AUX/IAA), auxin response factor (ARF), auxin responsive GH3 family protein (GH3) and small auxin-up RNA family protein (SAUR) (Fig. 5). The majority of them had lower expression under relatively low temperature, especially three ARF members (CL661.Contig9, CL274.Contig1, and CL12908.Contig6), which decreased sharply as the treated days lengthened. Only one AUX1, two AUX/IAA, one ARF, and one GH3 were up-regulated when treated with relatively low temperature (Fig. 5, Table S9).

The phytohormone JA and its derivative methyl jasmonate (MeJA) can affect the flower opening of *E. grandiflorum* [31], rice [32], *A. thaliana* [20]. The gene *BIGPETALp* (*BPEp*), which can be regulated by JA, may also play a role in JA-mediated petal growth [6]. In JA signal transduction pathway, most of genes such as Coronatine-insensitive protein 1 (*COI1*), jasmonate ZIM domain protein (*JAZ*), and *MYC2* were down-regulated in this study. Only two *JAZ* (Unigene43759 and CL8754.Contig3) genes and four *MYC2* (CL37.Contig1, CL6240.Contig1, CL3114.Contig2, and CL2658.Contig2) genes were up-regulated under 19 °C treatment (Fig. 5, Table S9), suggesting that these unigenes may function as positive regulators of flower opening in *O. fragrans* (Fig. 5, Table S9). Only six TGACG-sequence-specific DNA-binding protein (*TGA*) genes in salicylic acid (SA) signal transduction pathway were expressed differently under different temperatures and two of them (CL2000.Contig1 and CL225.Contig1) were increased after 19 °C treatment (Fig. 5, Table S9).

Nineteen unigenes in the gibberellin acid (GA) signaling pathway were also differentially expressed under different temperatures (Fig. 5). Five of six GID1 were down-regulated under relatively low temperature, with the exception of Unigene22649, which was nearly increased eight thousand times after 2 d treatment of 19 °C. Three DELLA and five PIF3 genes were decreased while two DELLA and three PIF3 genes were increased after relatively low temperature treatment (Fig. 5, Table S9). Only four of twenty unigenes including two PYR/PYL and two ABF (ABA responsive element binding factor) genes in ABA signal transduction pathway were up-regulated after 19 °C treatment. However, the expression of one ABF (CL12556.Contig1) was decreased by two hundred times under the relatively low temperature at 19 °C compared with that under 23 °C (Fig. 5, Table S9).

Most of the unigenes in cytokinin (CTK) signal transduction pathway were down-regulated under relatively low temperature including all *Arabidopsis* histidine phosphotransfer Protein (AHP) genes, and all B-ARR members, especially the *AHP* (CL11134.Contig2), which was decreased severely after 6 d treatment of 19 °C (Fig. 5). However, most of the A-ARR members (CL7866.Contig2, Unigene18524, and CL8358.Contig1) were up-regulated when treated by relatively low temperature (Fig. 5, Table S9).

The effect of ETH on flower opening may be dose-dependent, species-dependent or cultivar-dependent [19]. In ETH signal transduction pathway, the expression pattern of some ethylene receptor (ETR, Unigene14562), serine/threonine-protein kinase (CTR1, CL10648.Contig2), and ethylene insensitive 3 protein (EIN3, CL10633.Contig1) genes, were more complicated. For example, the expression level of Unigene14562 and CL10633.Contig1 were decreased when treated by 2 d of relatively low temperature, while they were increased when the treatment time was lengthened. Most of the genes in this pathway were down-regulated after the relatively low temperature’ treatment (Fig. 5, Table S9).

Cell elongation is also regulated by BR, which is a plant-specific steroid hormone and longitudinal cell expansion of BR mutants is greatly reduced in *A. thaliana* [33, 34]. Eight of fifteen unigenes in the BR signal transduction pathways were increased under 19 °C treatment, including all brassinosteroid receptor (BRI1), one BRI1 Associated receptor Kinase 1 (BSK), one TCH4, and one cyclin-D3 (CYCD3) (Fig. 5, Table S9). The expression level of BRI1, especially Unigene37674 and Unigene35684 were dramatically increased when treated with 19 °C. However, seven of fifteen unigenes in this signal pathway were decreased under 19 °C treatment. The expression level of BZR1/2 was eight-fold under 19 °C compared to 23 °C, and the expression level were continuously increased as the treatment time prolonged, suggesting the same role of BZR1/2 in petal development of *O. fragrans* and *G. hybrida.* Similarly, the expression level of *TCH4* (CL8777.Contig2) was enhanced by relatively low temperature.

### Identification of DEGs of transcription factors (TFs)

**Fig. 6:**
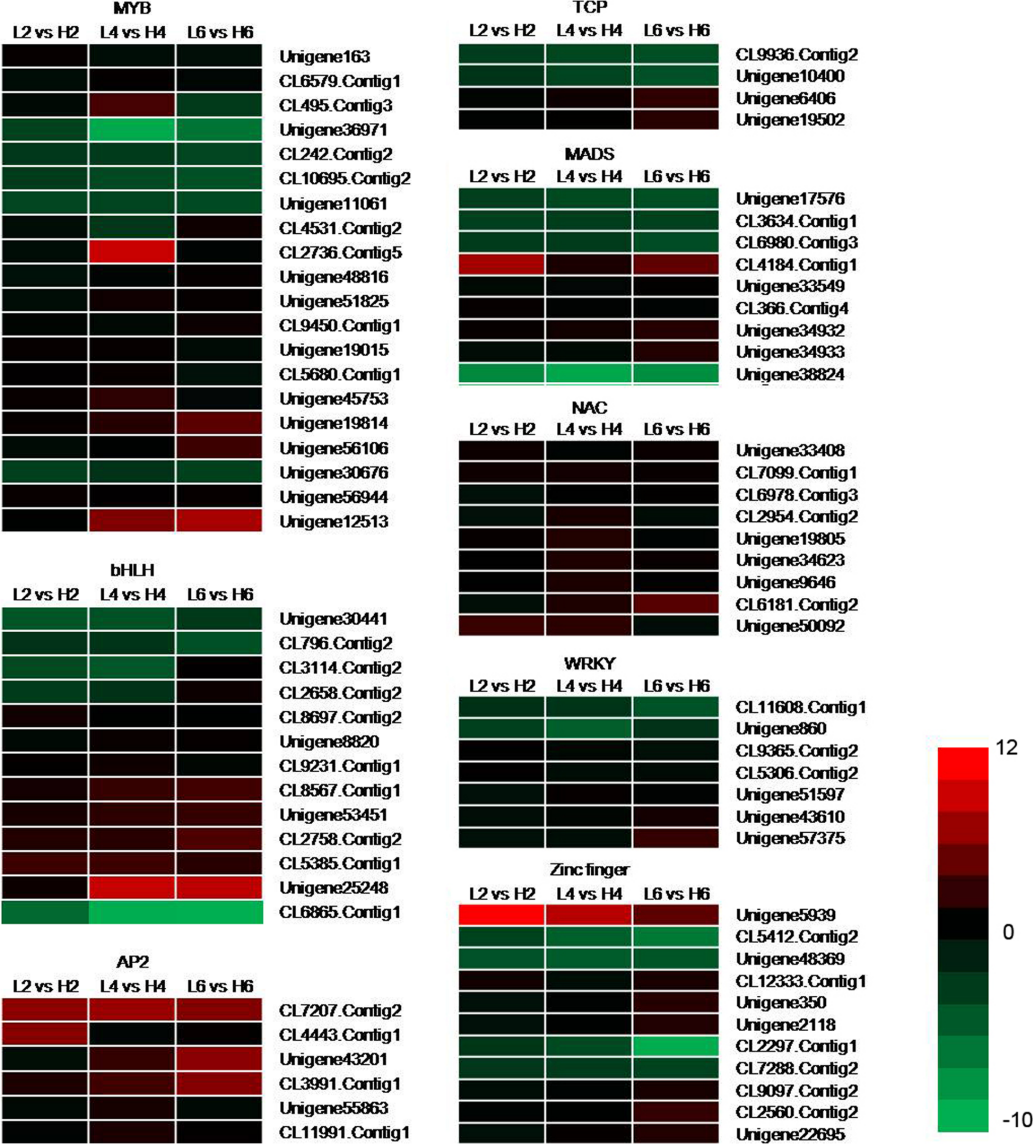
Heat maps of TF genes between the three comparisons (L2 vs H2, L4 vs H4, and L6 vs H6). Red rectangles represent the up-regulated unigenes, and green rectangles represent the down-regulated unigenes. All information for each gene list can be found in Table S10. L2, L4 and L6 respectively represents the samples from the plants under 19 °C treatment for 2 d, 4 d, and 6 d; H2, H4 and H6 respectively represents the samples from the plants under 23 °C treatment for 2 d, 4 d, and 6 d

Many TFs changed dramatically under different temperatures, so we improved the screening criteria. The TFs with a |log_2_Ratio| ≥ 2 in any comparison were analyzed further. 79 TFs which were divided into eight gene family (Fig. 6, Table S10) were selected for further analysis because they may be involved in petal cell expansion [5, 30]. In the MYB TF family, only five out of twenty genes (Unigene36971, CL242.Contig2, CL10695.Contig2, Unigene11061, and Unigene30676) were up-expressed under relatively low temperature. Overexpression of the *MYB62* gene results in a GA-deficient phenotype and can be partially alleviated with the application of exogenous GA [35]. In our study, we did not find the homologous unigenes to *MYB62* in *O. fragrans*, but some unigenes homologous to other gene family members, such as MYB4 (CL9450.Contig1) and MYB86 (Unigene19015), were expressed differently under different temperatures (Fig. 6, Table S10), indicating they may be involved in the regulation of relatively low temperature on flowering opening in *O. fragrans*.

In the bHLH TF family, three genes (Unigene30441, CL796.Contig2, and CL6865.Contig1) were up expressed after treated by 19 °C. Among them, especially the bHLH79, the expression level of bHLH79 isoform X2 (CL6865.Contig1) increased five hundred times when treated by 6 d of relatively low temperature. However, the expression level of bHLH 117 decreased sharply when exposed to 19 °C. None of the TF genes was up-regulated under relatively low temperature in the AP2 and NAC TF family. Plant-specific NAC family genes can regulate plant development, cell division, senescence, and responses to abiotic stress [36]. In recent research, the N-terminal binding domain of RhNAC2 could be bound to the promoter of *RhEXPA4* thus regulating of dehydration tolerance during the expansion of rose petals [37]. The expression of all the members of NAC family decreased under the relatively low temperature (Fig. 6, Table S10), indicating that the suppression of *NAC* genes may facilitate the expression of *EXP*s to promote the elongation of flower petals in *O. fragrans*.

In the TCP TF family, two genes (CL9936.Contig2, and Unigene10400) were up-expressed under relatively low temperature treatment. miRNAs, such as miRNA319a, have also been found to be involved in petal elongation by regulating *TCP4* which regulates AUX effects [38]. Therefore, *TCP4-like* gene (CL9936.Contig2, and Unigene10400) increased with relatively low temperature treatment, implying they may regulate the petal development in *O. fragrans* (Fig. 6, Table S10).

Four out of nine genes were up-regulated in the MADS TF family, especially the AGAMOUS gene, which is the C-function genes, and its expression increased significantly with the 19 °C treatment. In the WRKY TF family, only two genes (CL11608.Contig1, and Unigene860) were up-regulated under relatively low temperature. In Zinc finger TF family, four out of eleven genes’ expression increased when exposed to 19 °C. Generally speaking, only a small proportion of TFs were up-regulated under relatively low temperature.

### qRT-PCR analysis

**Fig. 7:**
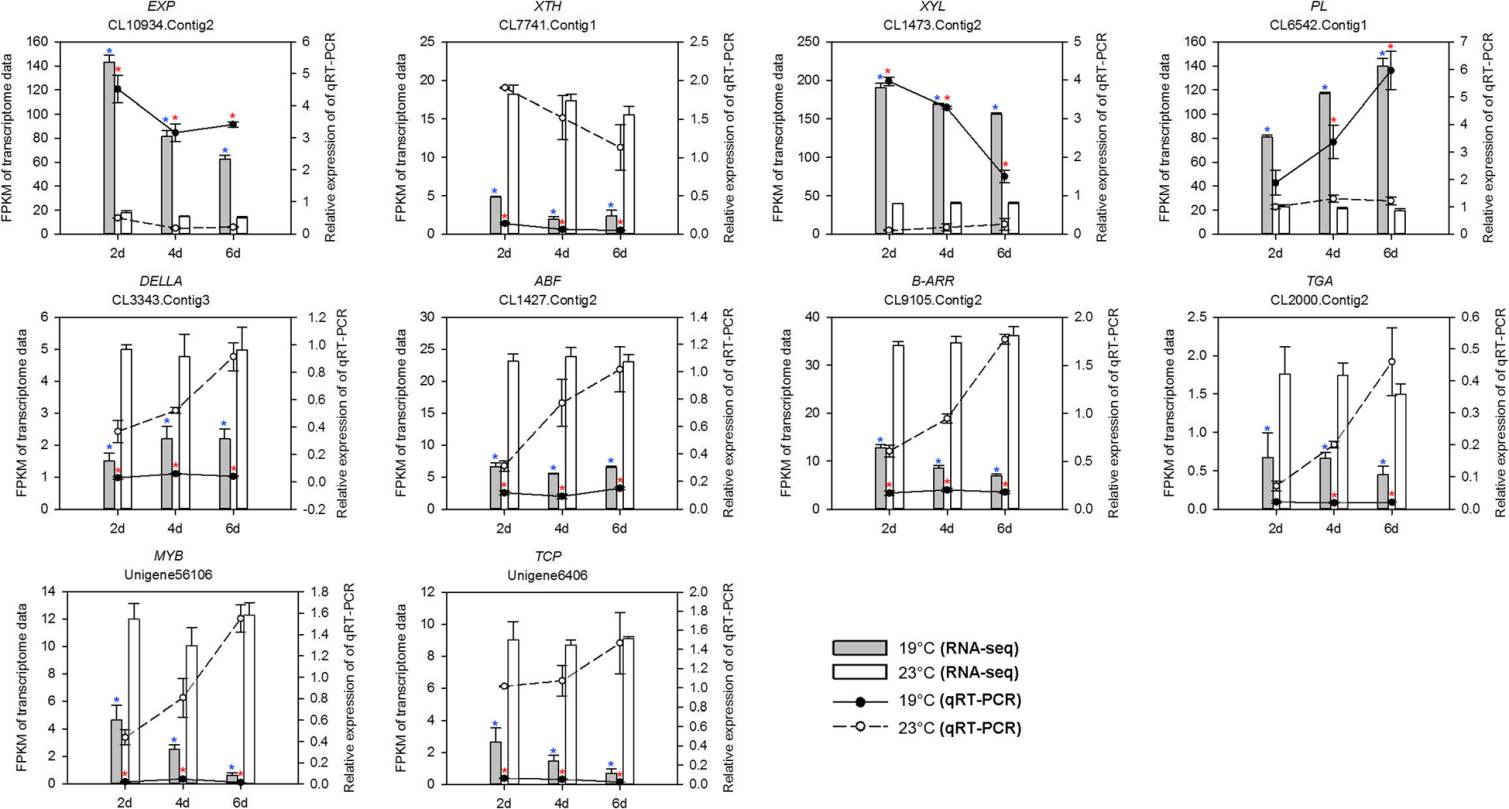
qRT-PCR and transcriptome results of the expression levels of 10 DEGs. *OfACT* was used as internal gene. Gene expression in 19 °C and 23 °C treatments from RNA-seq analysis. Data are showed as an average of three biological replicates and standard errors indicated by vertical bars. Compared to 23 °C treatment, relative expression level or FPKM of the same gene with 19 °C treatment at the same day is significantly different (*P* < 0.05, by Duncan’s multiple range test), which are marked with red asterisks in qRT-PCR analysis or blue asterisks in RNA-seq analysis

Ten differentially expressed unigenes comprising four genes involving in cell wall metabolism, four genes related to phytohormone signal transduction and two genes of TF were analyzed by qRT-PCR in order to confirm the results of RNA-Seq. All ten genes tested by qRT-PCR confirmed significant differential expression (*P* < 0.05) between 19 °C and 23 °C treatments (Fig. 7). Although fold changes of the ten genes between 19 °C and 23 °C treatments were not always similar in RNA-Seq and qRT-PCR results, the overall trend was consistent. Generally, the expression levels of CL10934.Contig2 (EXP), CL1473.Contig2 (XYL) and CL6542.Contig1 (PL) were significantly lower in 23 °C treatment than those in 19 °C treatment with qRT-PCR analysis, while the expression levels of other seven genes were higher in 23 °C treatment. Similarly, CL10934.Contig2, CL1473.Contig2 and CL6542.Contig1 were down-regulated, while other seven genes were up-regulated in 23 °C treatment, which verified the accuracy of RNA-Seq results.

## Discussion

The flowering time of once-flowering cultivars of *O. fragrans* is greatly affected by the relatively low temperature and they need a certain amount of low temperature accumulation at autumn before blooming [3]. The sensitivity difference to the requirement of relatively low temperature among different cultivars leads to the variation of flowering time [3]. In this study, 19 °C treatment in experimental condition for 6 d is enough for flower opening in the cultivar ‘Yanhong Gui’. The early flowering cultivar such as ‘Zao Yingui’ would blossom when the daily minimum temperature lower than 21 °C last for after 5–8 days, while the late blossoming cultivar, such as ‘Wan Yingui’, needs 5–8 days of the daily minimum temperature lower than 19 °C [39].

Flower opening is accompaning with cell division and cell expansion in petal cells [7]. are up-regulated during floral open process in sandersonia [40], *Mirabilis jalapa* [41], petunia [9], carnation [42], and wintersweet [10]. Similarly, during flower opening of *O. fragrans* under relatively low temperature, four *α-EXP* were up-regulated in this study (Fig. 4, Table S8). It was also reported that β-EXP is more efficient in disrupting cell wall polymers than α-EXP [41], however, one *β-EXP* was down-regulated under 19 °C treatment. The result is coincidence with the results in rose [30], suggesting that β-EXP may be involved in petal development in a special manner different from α-EXP. As to XTH, four rose *XTH* genes (*RbXTH3*, *RbXTH5*, *RbXTH6*, and *RbXTH12*) were up-regulated during flower opening and associated with petal movement [43]. Rice *OsXTH12* gene can cause rice glume-unclosing after anthesis under high temperature conditions [44]. In our experiment, we cannot found the homologous genes of *XTH3*, *XTH5*, *XTH6* and *XTH12*, but other family members were discovered. Most of *XTH* genes were up-regulated under relatively low temperature, indicating that they may be involved in the flower opening process of *O. fragrans* and different family members may have different functions.

These cell wall metabolism processes were also influenced by multiple kinds of phytohormones via transcription factors [19]. The expression patterns of phytohormone pathway genes are different under different temperatures treated by different days in our research. In *A. thaliana*, both the AUX and JA signal transduction function synergistically during petal development [45]. And ARF8 can interacts with BIGPETALp (BPEp) to regulate cell expansion in petals [6] and can trigger the expression of *MYB21* and *MYB24* to induce the production of JA to promote petal growth [45]. However, most DEGs involved in AUX and JA signal pathways were down-regulated (Fig. 5, Table S9), suggesting that the regulation of relatively low temperature on flower opening in *O. fragrans* doesn’t depend on AUX and JA signaling.

GA can regulate seed germination, stem elongation, petal growth and flowering [46], and ABA has an antagonistically role such as in floral transition and fruit development [47]. The same circumstance occurred in the *G. hybrida* petal growth [22]. Compared to unigenes involved in ABA signal transduction pathway, there were much more unigenes in GA signal transduction pathway which increased sharply under the treatment of 19 °C. This result was consistent with the result in rose [30]. However, whether this kind of antagonistic role exists in petal growth of *O. fragrans* still needs further demonstration.

ETH can promote flower opening in carnation, *Phalaenopsis* orchids, and petunia, but it inhibits flower opening in several rose cultivars [19]. ETH can induce the expression of DELLA genes via EIN3–3 when it functions as inhibitors during flower opening in rose [22]. In our study, EIN3 was down-regulated under relatively low temperature (Fig. 5, Table S9), suggesting that ETH may function negatively on flower opening in response to relatively low temperature in *O. fragrans*.

In *G. hybrida*, BL shows more efficient effect on lengthening the cells in the middle and basal regions of petals than GA [21]. But the expression level of BES1/BZR1 was decreased after only 0.5 h BR treatment, indicating its vital role in petal cell expansion [21]. In our study, expression level of the homologous unigene of BZR1 (Unigene45694), decreased dramatically when exposed to relatively low temperature (Fig. 5, Table S9), suggesting the same role in petal development of *O. fragrans* as that in *G. hybrida*.

## Conclusion

The comprehensive transcriptomic dataset relating to flower opening of *O. fragrans* under relatively low temperature (19 °C) treatment was performed using RNA-seq technology, and a group of DEGs, which may regulate petal growth through pairwise comparison analysis of DEGs between different temperature treatments, had been identified, including unigenes involved in cell wall metabolism, phytohormone signal transduction pathway, and TFs. Overall, the resources generated in this study would lay foundation on fully revealing the molecular mechanism of relatively-low temperature-regulated flower opening of *O. fragrans* and provide theoretical reference for the prediction and regulation of flowering time and genetic modification in *O. fragrans*. Our future research will be focused on understanding the biological function of these candidate important DEGs and revealing how these DEGs response to relatively low temperature in *O. fragrans*.

## Methods

### Plant materials and treatments

Plants of *O. fragrans* ‘Yanhong Gui’ were potted and grown in the resource nursery of Zhejiang Agriculture and Forestry University. All plant materials were owned by Zhejiang Agriculture and Forestry University. The development of flower opening process are classified into S1-S6 as described in this paper [3]. When the floral development stage was at S1, potted plants were moved into the growth cabinet with the temperature of 19 °C (relatively low temperature) or 23 °C (relative high temperature), respectively. The relative humidity was about 50–60% and the photoperiod was 12 h light/12 h dark regime with an illumination of 80 μmol·m^− 2^·s^− 1^. Petals were harvested every 2 days after treatment until the developmental stage of plants under 19 °C condition reached S2. The samples were named as H2, H4, H6 (treated for 2 d, 4 d, and 6 d under 23 °C treatment, respectively) and L2, L4, L6 (treated by 2 d, 4 d, and 6 d under 19 °C treatment, respectively). All samples were collected at 10:00 am in order to avoid the influence of diurnal rhythm. Three biological replicate samples were collected to generate 18 samples in total. In addition, petals at different developmental stages (S1-S6) were respectively sampled for SEM and TEM analysis.

### SEM and TEM analysis

According to previous studies [25, 48], SEM and TEM were carried out and were observed and photographed in Hitachi Model TM-1000 SEM and Model H-7650 respectively in Zhejiang University. Cell size of adaxial and abaxial petal epidermal cells, and vacuole size were manually measured using Image J software (http://rsb.info.nih.gov/ij/, NIH, MD, USA).

### RNA extraction, cDNA library preparation and sequencing

RNA isolation and RNA purification of 18 samples were carried out as described previously [49]. The cDNA library preparation and sequencing of 18 samples of *O. fragrans* were respectively performed as described previously [50] for RNA-seq analysis. What’s more, a RNA pool mixed from 18 RNA samples was used to construct a cDNA library for reference transcriptome sequencing. The Illumina sequencing was performed at the Beijing Genomics Institute (BGI) (Shenzhen, China) (http://www.genomics.cn/index.php) according to the manufacturer’s instructions (Illumina Inc., San Diego, CA, USA). A total of 18 sets of raw reads were obtained for RNA-seq analysis, corresponding to H2–1, H2–2, H2–3, H4–1, H4–2, H4–3, H6–1, H6–2, H6–3, L2–1, L2–2, L2–3, L4–1, L4–2, L4–3, L6–1, L6–2, and L6–3, while raw reads were obtained for reference transcriptome corresponding to ALL.

### Transcriptome data processing and analysis

Raw reads were cleaned by removing the sequence of adaptor, high content of unknown bases and low-quality reads before downstream analysis to decrease data noise. As *O. fragrans* did not have an appropriate reference genome sequence, the Trinity method [51] was used to de novo assemble all clean reads and the Tgicl method [52] was used to cluster the transcripts to obtain non-redundant unigenes. The assembled unigene sequences were aligned by Blastn [53] to nucleotide database NT, aligned by Blastx [53] or Diamond [54] to protein databases NR, Swiss-Prot, KEGG, and KOG, and aligned by InterProScan5 [55] to protein database InterPro to get the annotations. With the NR annotation, GO annotations of unigenes were obtained using Blast2GO software [56]. FPKM (reads mapped per 1000 bp per million sequenced reads) method was used in calculated expression level [57]. Comparisons of FPKM between samples treated under different temperatures for the same number of days (L2 vs H2, L4 vs H4, and L6 vs H6 respectively) were performed for each unigene. To identify the DEGs in the two samples, the Audic and Claverie method was employed [58]. Unigene with a *P* value ≤0.01 and a |log_2_Ratio| ≥ 1 were considered significant DEGs. All DEGs were mapped to each term of the GO and KEGG databases. For the analysis of transcription factors, |log_2_Ratio| ≥ 2 were marked to be significantly different between the two samples.

### Quantitative real-time PCR (qRT-PCR) analysis

The extraction of total RNA, first-strand cDNA synthesis, and qRT-PCR were performed as previously described [49]. Ten DEGs were randomly chosen and the primer sequences were listed in Table S11. Gene relative expression levels of those ten genes were normalized relatively to the expression level of an internal control *OfACT* [59] using the 2^-△Ct^ method.

### Statistical analysis

The statistical analysis of the size of adaxial petal epidermal cells, abaxial petal epidermal cells, and vacuole, as well as relative expression level and FPKM analysis was performed by one-way analysis of variance (ANOVA) using SPSS software version 18.0 (SPSS Inc., Chicago, IL, USA). Duncan’s multiple range test was employed and differences of *P* < 0.05 were considered significant.

## Supplementary information

**Additional file 1: Figure S1.** A species-based distribution of BLASTX matches for unigenes from the reference transcriptome of *Osmanthus fragrans*. We used all the plant proteins in the NCBI NR database to perform the homology search and for each sequence we selected the closest match for analysis.

**Additional file 2: Figure S2.** GO classification of unigenes from the reference transcriptome of *Osmanthus fragrans*. Results are summarized under three main GO categories: biological process, cellular component and molecular function. The right x-axis indicates the number of genes in the same category.

**Additional file 3: Figure S3.** KOG classification of *Osmanthus fragrans* unigenes from the reference transcriptome. From a total of 96,920 de novo assembled unigenes, 43,496 transcripts with significant homologies in the KOG database were classified into 25 KOG categories.

**Additional file 4: Table S1.** The size of adaxial petal epidermal cells, abaxial petal epidermal cells, and vacuole of adaxial petal epidermal cells in sweet osmanthus flowers at different developmental stages

**Additional file 5: Table S2.** Summary of Illumina transcriptomic sequencing.

**Additional file 6: Table S3.** Summary of assembly quality of transcripts and unigenes.

**Additional file 7: Table S4.** Annotation results of the assembled unigenes from the reference transcriptome against public databases.

**Additional file 8: Table S5.** KEGG pathway annotation of *Osmanthus fragrans* unigenes obtained from the reference transcriptome. There were 40,764 unigenes mapped into 134 KEGG pathways.

**Additional file 9: Table S6.** Number of total clean reads of the 18 samples mapped to reference sequences.

**Additional file 10: Table S7.**Pearson correlation coefficients of six samples each with three biological replicates.

**Additional file 11: Table S8.** DEGs involved in the cell wall metabolism under different temperatures treated by different days in *Osmanthus fragrans.*

**Additional file 12: Table S9.** DEGs involved in the phytohormone signal transduction pathway under different temperatures treated by different days in *Osmanthus fragrans.*

**Additional file 13: Table S10.** DEGs involved in TFs under different temperatures treated by different days in *Osmanthus fragrans.*

**Additional file 14: Table S11.** Primer sequences and amplicon characteristics.

## Data Availability

The RNA-Seq datasets used in the current study are available on the NCBI Short Read Archive Project - PRJNA642321 (http://www.ncbi.nlm.nih.gov/bioproject/PRJNA642321). All RNA-seq reads generated by this study are publicly available at the NCBI SRA under accession numbers SRR12109992-SRR12110009.

## Abbreviations

ABA: Abscisic acid
ARF: Auxin response factor
AHP: *Arabidopsis* histidine phosphotransfer protein
AUX: Auxin
AUX/IAA: Auxin-induced protein/auxin-responsive protein
AUX1: Auxin transporter protein
BPEp: BIGPETALp
BR: Brassinosteroid
CES: Cellulose synthase
COI1: Coronatine-insensitive protein 1
CYCD3: Cyclin-D3
CTK: Cytokinin
CTR1: Serine/threonine-protein kinase
DEGs: Differentially expressed genes
EIN3: Ethylene insentitive 3 protein
ETH: Ethylene
ETR: Ethylene receptor
EXP: Expansin
FPKM: Reads mapped per 1000 bp per million sequenced reads
GA: Gibberellin
GH3: Auxin responsive GH3 family protein
IAA: Indole-3-acetic acid
IBA: Indole-3-butyric acid
JA: Jasmonic acid
JAZ: Jasmonate ZIM domain protein
KEGG: Kyoto Encyclopedia of Genes and Genomes
KOG: Eukaryotic Orthologous Groups of proteins
MeJA: Methyl jasmonate
NIP: Nodulin-26-like intrinsic membrane protein
NR: Non-Redundant Protein Sequence Database
NT: Nucleotide Sequence Database
GA: Gibberellin acid
GO: Gene Ontology
PE: Pectin esterase
PG: Polygalacturonase
PIP: Plasma membrane intrinsic protein
PL: Pectate lyase
qRT-PCR: Quantitative real-time PCR
SA: Salicylic acid
SAUR: Small auxin-up RNA family protein
SEM: Scanning electron microscopy
SIP: Small basic intrinsic protein
TGA: TGACG-sequence-specific DNA-binding protein
TEM: Transmission electron microscopy
TIP: Tonoplast intrinsic protein
XTH: Xyloglucan endotransglycosylase/hydrolase
XYL: Xylosidase
