# Supplementary figures and images for: Transcriptomic analysis of flower opening response to relatively low temperatures in Osmanthus fragrans
Source: BMC Plant Biol. 2020 Jul 16;20:337. doi: 10.1186/s12870-020-02549-3 (PMC7367400; doi:10.1186/s12870-020-02549-3)

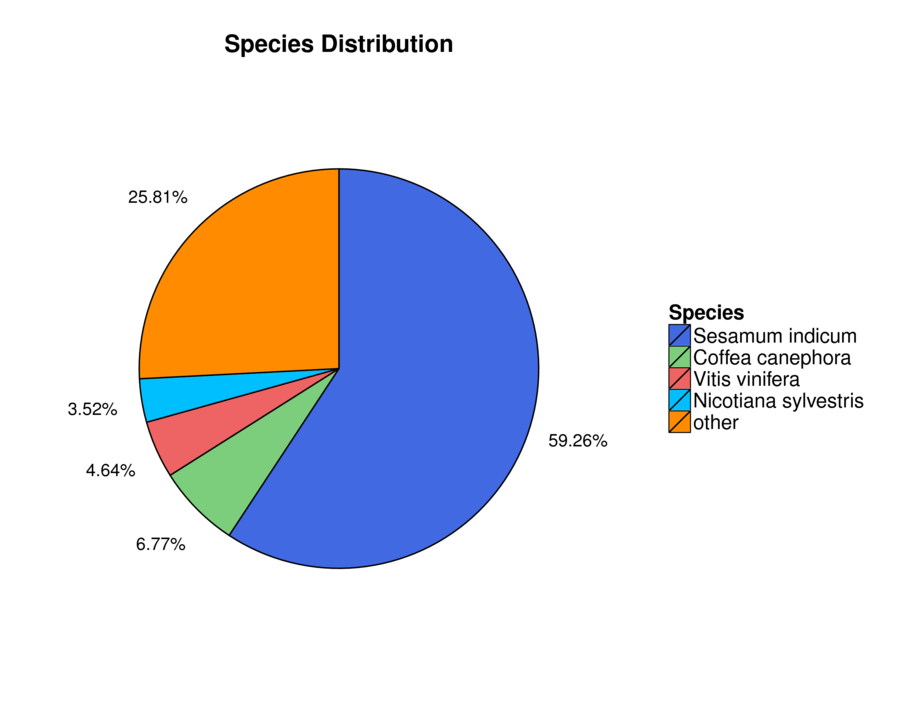

Supplement: Supplementary file 1 — Additional file 1: Figure S1. A species-based distribution of BLASTX matches for unigenes from the reference transcriptome of Osmanthus fragrans. We used all the plant proteins in the NCBI NR database to perform the homology search and for each sequence we selected the closest match for analysis. [file 12870_2020_2549_MOESM1_ESM.png]

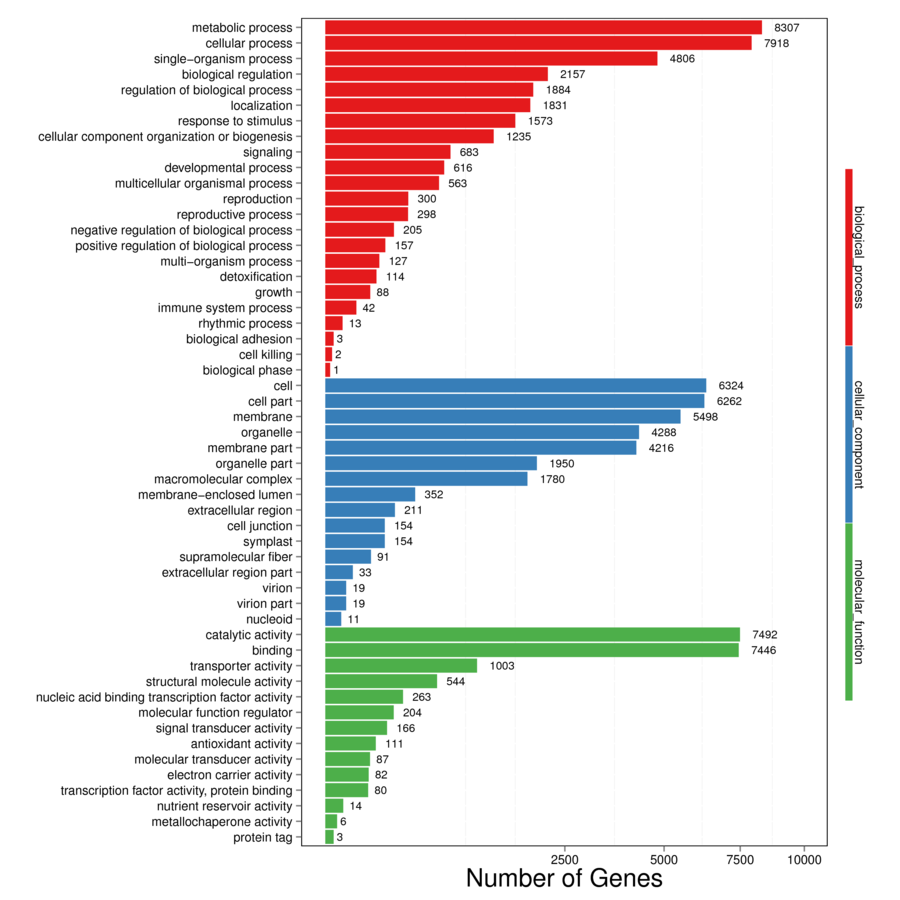

Supplement: Supplementary file 2 — Additional file 2: Figure S2. GO classification of unigenes from the reference transcriptome of Osmanthus fragrans. Results are summarized under three main GO categories: biological process, cellular component and molecular function. The right x-axis indicates the number of genes in the same category. [file 12870_2020_2549_MOESM2_ESM.png]

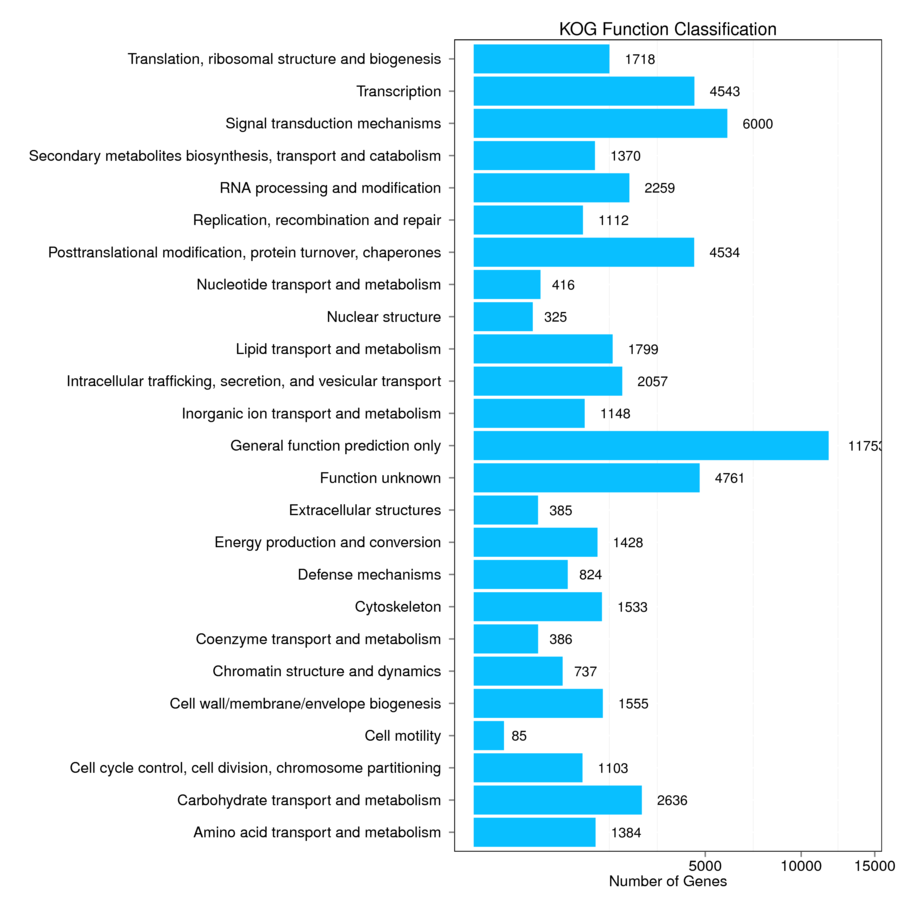

Supplement: Supplementary file 3 — Additional file 3: Figure S3. KOG classification of Osmanthus fragrans unigenes from the reference transcriptome. From a total of 96,920 de novo assembled unigenes, 43,496 transcripts with significant homologies in the KOG database were classified into 25 KOG categories. [file 12870_2020_2549_MOESM3_ESM.png]
