# Supplementary material for: Transcriptomic analysis of flower opening response to relatively low temperatures in Osmanthus fragrans
Source: BMC Plant Biol. 2020 Jul 16;20:337. doi: 10.1186/s12870-020-02549-3 (PMC7367400; doi:10.1186/s12870-020-02549-3)
Supplement: Supplementary file 4 — Additional file 4: Table S1. The size of adaxial petal epidermal cells, abaxial petal epidermal cells, and vacuole of adaxial petal epidermal cells in sweet osmanthus flowers at different developmental stages [file 12870_2020_2549_MOESM4_ESM.docx]

Table S1 The size of adaxial petal epidermal cells, abaxial petal epidermal cells, and vacuole of adaxial petal epidermal cells in sweet osmanthus flowers at different developmental stages

| Developmental stages | Cell size (μm^2^) | | Vacuole size (μm^2^) |
| --- | --- | --- | --- |
|  | Adaxial petal epidermal cells | Abaxial petal epidermal cells |  |
| S1 | 226.42±35.55 c | 239.70±25.94 c | 179.85±6.05 d |
| S2 | 311.41±34.22 bc | 255.17±13.05 c | 346.26±38.77 c |
| S3 | 457.12±43.91 ab | 467.50±50.36 c | 401.08±16.71 bc |
| S4 | 572.08±19.05 a | 998.58±48.29 b | 488.05±18.86 bc |
| S5 | 578.05±40.72 a | 1376.50±85.43 a | 508.90±57.15 b |
| S6 | 495.43±36.55 ab | 1012.22±103.48 b | 694.47±22.53 a |

Note: Values are presented as the mean ± SE. Letters indicate significant differences between the respective values (*P* < 0.05, by Duncan’s multiple range test). S1, the outer bud scales unfurled and the inner bud scales still furled; S2, the bud became globular-shaped and the inside bracts covering the inﬂorescence was visible; S3, the inﬂorescence burst through bracts and the ﬂorets closely crowded; S4, initial ﬂowering stage; S5, full ﬂowering stage; S6, pollen-scattered stage.
