# Supplementary material for: Transcriptomic analysis of flower opening response to relatively low temperatures in Osmanthus fragrans
Source: BMC Plant Biol. 2020 Jul 16;20:337. doi: 10.1186/s12870-020-02549-3 (PMC7367400; doi:10.1186/s12870-020-02549-3)
Supplement: Supplementary file 5 — Additional file 5: Table S2. Summary of Illumina transcriptomic sequencing. [file 12870_2020_2549_MOESM5_ESM.doc]

Table S2 Summary of Illumina transcriptomic sequencing

| Sample | Raw reads number | Clean data size (bp) | Clean reads number | Clean data rate (%) |
| --- | --- | --- | --- | --- |
| ALL | 164,753,984 | 11,030,736,600 | 110,307,366 | 66.95 |
| L2-1 | 23,640,350 | 1,181,657,500 | 23,633,150 | 99.96 |
| L2-2 | 23,810,513 | 1,190,095,050 | 23,801,901 | 99.96 |
| L2-3 | 23,694,416 | 1,184,400,300 | 23,688,006 | 99.97 |
| L4-1 | 23,584,245 | 1,178,884,200 | 23,577,684 | 99.97 |
| L4-2 | 23,675,804 | 1,183,479,400 | 23,669,588 | 99.97 |
| L4-3 | 24,137,014 | 1,206,149,150 | 24,122,983 | 99.94 |
| L6-1 | 23,605,194 | 1,180,007,700 | 23,600,154 | 99.97 |
| L6-2 | 24,136,714 | 1,206,554,250 | 24,131,085 | 99.97 |
| L6-3 | 24,136,970 | 1,206,414,600 | 24,128,292 | 99.96 |
| H2-1 | 23,739,427 | 1,186,702,750 | 23,734,055 | 99.97 |
| H2-2 | 24,137,008 | 1,206,446,550 | 24,128,931 | 99.96 |
| H2-3 | 24,136,915 | 1,206,548,000 | 24,130,960 | 99.97 |
| H4-1 | 23,724,418 | 1,185,600,200 | 23,712,004 | 99.94 |
| H4-2 | 24,136,770 | 1,206,413,200 | 24,128,264 | 99.96 |
| H4-3 | 24,136,916 | 1,206,494,800 | 24,129,896 | 99.97 |
| H6-1 | 23,689,085 | 1,184,139,800 | 23,682,796 | 99.97 |
| H6-2 | 24,137,031 | 1,206,533,000 | 24,130,660 | 99.97 |
| H6-3 | 24,136,860 | 1,206,461,850 | 24,129,237 | 99.96 |

Note: L2, L4 and L6 respectively represents the samples from the plants under 19°C treatment for 2 d, 4 d, and 6 d; H2, H4 and H6 respectively represents the samples from the plants under 23°C treatment for 2 d, 4 d, and 6 d; ALL represents the reference transcriptome from a RNA mixed pool of three biological replicate samples of H2, H4, H6, L2, L4, and L6.
