# Supplementary material for: Transcriptomic analysis of flower opening response to relatively low temperatures in Osmanthus fragrans
Source: BMC Plant Biol. 2020 Jul 16;20:337. doi: 10.1186/s12870-020-02549-3 (PMC7367400; doi:10.1186/s12870-020-02549-3)
Supplement: Supplementary file 6 — Additional file 6: Table S3. Summary of assembly quality of transcripts and unigenes. [file 12870_2020_2549_MOESM6_ESM.doc]

Table S3 Summary of assembly quality of transcripts and unigenes

| Assembly type | Total number | Total length | Mean length | N50 | N90 | GC (%) |
| --- | --- | --- | --- | --- | --- | --- |
| Transcripts | 152,247 | 114,487,589 | 751 | 1,361 | 284 | 40.48 |
| Unigenes | 96,920 | 84,668,764 | 873 | 1,488 | 338 | 40.41 |

Note: the assembly transcripts are ordered from longest to shortest, and N50 is the length of transcripts that are no less than 50% the total length, N90 is the length of transcripts that are no less than 90% the total length.**a**
