# Supplementary material for: Transcriptomic analysis of flower opening response to relatively low temperatures in Osmanthus fragrans
Source: BMC Plant Biol. 2020 Jul 16;20:337. doi: 10.1186/s12870-020-02549-3 (PMC7367400; doi:10.1186/s12870-020-02549-3)
Supplement: Supplementary file 7 — Additional file 7: Table S4. Annotation results of the assembled unigenes from the reference transcriptome against public databases. [file 12870_2020_2549_MOESM7_ESM.doc]

Table S4 Annotation results of the assembled unigenes from the reference transcriptome against public databases

| Database | Number of annotated unigenes | Percentage (%) |
| --- | --- | --- |
| NR | 57,721 | 59.56 |
| NT | 41,181 | 42.49 |
| Swiss-Prot | 37,284 | 38.47 |
| KEGG | 40,764 | 42.06 |
| KOG | 43,496 | 44.88 |
| InterPro | 46,405 | 47.88 |
| GO | 16,014 | 16.52 |
| Intersection | 8,150 | 8.41 |
| Overall | 61,654 | 63.61 |
