# Supplementary material for: Transcriptomic analysis of flower opening response to relatively low temperatures in Osmanthus fragrans
Source: BMC Plant Biol. 2020 Jul 16;20:337. doi: 10.1186/s12870-020-02549-3 (PMC7367400; doi:10.1186/s12870-020-02549-3)
Supplement: Supplementary file 9 — Additional file 9: Table S6. Number of total clean reads of the 18 samples mapped to reference sequences. [file 12870_2020_2549_MOESM9_ESM.doc]

Table S6 Number of total clean reads of the 18 samples mapped to reference sequences

| Samples | Total mapped reads percentage (%) | Unique match percentage (%) | Multi-position match percentage (%) | Total unmapped reads percentage (%) |
| --- | --- | --- | --- | --- |
| L2-1 | 86.93 | 54.51 | 32.42 | 13.07 |
| L2-2 | 87.05 | 54.55 | 32.50 | 12.95 |
| L2-3 | 86.92 | 54.57 | 32.36 | 13.08 |
| L4-1 | 87.71 | 55.77 | 31.93 | 12.29 |
| L4-2 | 87.81 | 55.80 | 32.01 | 12.19 |
| L4-3 | 87.90 | 55.74 | 32.16 | 12.10 |
| L6-1 | 88.16 | 56.25 | 31.91 | 11.84 |
| L6-2 | 88.44 | 56.28 | 32.16 | 11.56 |
| L6-3 | 88.26 | 56.29 | 31.96 | 11.74 |
| H2-1 | 86.54 | 53.89 | 32.65 | 13.46 |
| H2-2 | 86.78 | 54.09 | 32.69 | 13.22 |
| H2-3 | 86.66 | 53.90 | 32.76 | 13.34 |
| H4-1 | 86.39 | 53.76 | 32.63 | 13.61 |
| H4-2 | 86.66 | 53.86 | 32.80 | 13.34 |
| H4-3 | 86.43 | 53.70 | 32.73 | 13.57 |
| H6-1 | 86.43 | 53.96 | 32.47 | 13.57 |
| H6-2 | 86.64 | 53.99 | 32.65 | 13.36 |
| H6-3 | 86.69 | 53.92 | 32.77 | 13.31 |

Note: L2, L4 and L6 respectively represents the samples from the plants under 19°C treatment for 2 d, 4 d, and 6 d; H2, H4 and H6 respectively represents the samples from the plants under 23°C treatment for 2 d, 4 d, and 6 d.
