# Supplementary material for: Transcriptomic analysis of flower opening response to relatively low temperatures in Osmanthus fragrans
Source: BMC Plant Biol. 2020 Jul 16;20:337. doi: 10.1186/s12870-020-02549-3 (PMC7367400; doi:10.1186/s12870-020-02549-3)
Supplement: Supplementary file 10 — Additional file 10: Table S7.Pearson correlation coefficients of six samples each with three biological replicates. [file 12870_2020_2549_MOESM10_ESM.doc]

Table S7 Pearson correlation coefficients of six samples each with three biological replicates

| Sample | L2-1 | L2-2 | L2-3 | L4-1 | L4-2 | L4-3 | L6-1 | L6-2 | L6-3 | H2-1 | H2-2 | H2-3 | H4-1 | H4-2 | H4-3 | H6-1 | H6-2 | H6-3 |
| --- | --- | --- | --- | --- | --- | --- | --- | --- | --- | --- | --- | --- | --- | --- | --- | --- | --- | --- |
| L2-1 | 1.00 | 0.99 | 1.00 | 0.87 | 0.88 | 0.88 | 0.78 | 0.79 | 0.79 | 0.72 | 0.73 | 0.72 | 0.69 | 0.69 | 0.70 | 0.68 | 0.68 | 0.68 |
| L2-2 | 0.99 | 1.00 | 1.00 | 0.85 | 0.85 | 0.87 | 0.74 | 0.75 | 0.76 | 0.68 | 0.70 | 0.71 | 0.65 | 0.67 | 0.67 | 0.64 | 0.65 | 0.65 |
| L2-3 | 1.00 | 1.00 | 1.00 | 0.87 | 0.87 | 0.88 | 0.77 | 0.78 | 0.78 | 0.71 | 0.72 | 0.71 | 0.68 | 0.68 | 0.69 | 0.67 | 0.67 | 0.67 |
| L4-1 | 0.87 | 0.85 | 0.87 | 1.00 | 1.00 | 0.99 | 0.85 | 0.85 | 0.84 | 0.71 | 0.69 | 0.62 | 0.69 | 0.66 | 0.65 | 0.68 | 0.64 | 0.62 |
| L4-2 | 0.88 | 0.85 | 0.87 | 1.00 | 1.00 | 0.99 | 0.86 | 0.86 | 0.85 | 0.72 | 0.70 | 0.63 | 0.70 | 0.67 | 0.65 | 0.69 | 0.65 | 0.63 |
| L4-3 | 0.88 | 0.87 | 0.88 | 0.99 | 0.99 | 1.00 | 0.84 | 0.85 | 0.84 | 0.69 | 0.68 | 0.63 | 0.66 | 0.65 | 0.64 | 0.65 | 0.63 | 0.62 |
| L6-1 | 0.78 | 0.74 | 0.77 | 0.85 | 0.86 | 0.84 | 1.00 | 0.99 | 0.99 | 0.74 | 0.71 | 0.62 | 0.74 | 0.68 | 0.67 | 0.72 | 0.67 | 0.64 |
| L6-2 | 0.79 | 0.75 | 0.78 | 0.85 | 0.86 | 0.85 | 0.99 | 1.00 | 1.00 | 0.72 | 0.69 | 0.62 | 0.71 | 0.67 | 0.66 | 0.70 | 0.66 | 0.63 |
| L6-3 | 0.79 | 0.76 | 0.78 | 0.84 | 0.85 | 0.84 | 0.99 | 1.00 | 1.00 | 0.71 | 0.69 | 0.62 | 0.70 | 0.67 | 0.65 | 0.69 | 0.65 | 0.63 |
| H2-1 | 0.72 | 0.68 | 0.71 | 0.71 | 0.72 | 0.69 | 0.74 | 0.72 | 0.71 | 1.00 | 0.99 | 0.94 | 1.00 | 0.98 | 0.97 | 0.99 | 0.97 | 0.96 |
| H2-2 | 0.73 | 0.70 | 0.72 | 0.69 | 0.70 | 0.68 | 0.71 | 0.69 | 0.69 | 0.99 | 1.00 | 0.97 | 0.98 | 0.99 | 0.99 | 0.98 | 0.99 | 0.98 |
| H2-3 | 0.72 | 0.71 | 0.71 | 0.62 | 0.63 | 0.63 | 0.62 | 0.62 | 0.62 | 0.94 | 0.97 | 1.00 | 0.92 | 0.97 | 0.98 | 0.93 | 0.97 | 0.98 |
| H4-1 | 0.69 | 0.65 | 0.68 | 0.69 | 0.70 | 0.66 | 0.74 | 0.71 | 0.70 | 1.00 | 0.98 | 0.92 | 1.00 | 0.98 | 0.97 | 0.99 | 0.97 | 0.96 |
| H4-2 | 0.69 | 0.67 | 0.68 | 0.66 | 0.67 | 0.65 | 0.68 | 0.67 | 0.67 | 0.98 | 0.99 | 0.97 | 0.98 | 1.00 | 1.00 | 0.99 | 0.99 | 0.99 |
| H4-3 | 0.70 | 0.67 | 0.69 | 0.65 | 0.65 | 0.64 | 0.67 | 0.66 | 0.65 | 0.97 | 0.99 | 0.98 | 0.97 | 1.00 | 1.00 | 0.98 | 0.99 | 0.99 |
| H6-1 | 0.68 | 0.64 | 0.67 | 0.68 | 0.69 | 0.65 | 0.72 | 0.70 | 0.69 | 0.99 | 0.98 | 0.93 | 0.99 | 0.99 | 0.98 | 1.00 | 0.99 | 0.97 |
| H6-2 | 0.68 | 0.65 | 0.67 | 0.64 | 0.65 | 0.63 | 0.67 | 0.66 | 0.65 | 0.97 | 0.99 | 0.97 | 0.97 | 0.99 | 0.99 | 0.99 | 1.00 | 1.00 |
| H6-3 | 0.68 | 0.65 | 0.67 | 0.62 | 0.63 | 0.62 | 0.64 | 0.63 | 0.63 | 0.96 | 0.98 | 0.98 | 0.96 | 0.99 | 0.99 | 0.97 | 1.00 | 1.00 |
